# Status of ALS Treatment, Insights into Therapeutic Challenges and Dilemmas

**DOI:** 10.3390/jpm12101601

**Published:** 2022-09-28

**Authors:** Mohammed Khamaysa, Pierre-François Pradat

**Affiliations:** 1 Laboratoire d’Imagerie Biomédicale, Sorbonne Université, CNRS, INSERM, 75006 Paris, France; 2 Centre Référent SLA, Département de Neurologie, AP-HP, Hôpital Pitié-Salpêtrière, 75013 Paris, France; 3 Northern Ireland Centre for Stratified Medicine, Biomedical Sciences Research Institute, Ulster University, C-TRIC, Altnagelvin Hospital, Derry-Londonderry BT47 6SB, UK

**Keywords:** riluzole, preclinical models, protein homeostasis inductors, gene targeted strategies, ALS trials platforms

## Abstract

Amyotrophic lateral sclerosis (ALS) is an extremely heterogeneous disease of motor neurons that eventually leads to death. Despite impressive advances in understanding the genetic, molecular, and pathological mechanisms of the disease, the only drug approved to date by both the FDA and EMA is riluzole, with a modest effect on survival. In this opinion view paper, we will discuss how to address some challenges for drug development in ALS at the conceptual, technological, and methodological levels. In addition, socioeconomic and ethical issues related to the legitimate need of patients to benefit quickly from new treatments will also be addressed. In conclusion, this brief review takes a more optimistic view, given the recent approval of two new drugs in some countries and the development of targeted gene therapies.

## 1. Introduction

Amyotrophic lateral sclerosis (ALS) was first described by Jean-Martin Charcot in 1869 as an inexorably progressive disease primarily associated with degeneration of upper and lower motor neurons [1]. The early 1990s was an important turning point in the study of ALS when the positive results of a large therapeutic trial of riluzole, an anti-glutamatergic agent, were published, showing a prolongation of patient survival [2]. As a result, riluzole was approved by the FDA as the first drug for ALS. To date, it is the only therapeutic agent approved in both the United States and Europe with a modest effect, prolonging patients’ lives by only a few months without significantly improving functional deterioration [3]. Edaravone (Radicava^TM^) was approved by the Food and Drugs Administration (FDA) in 2017 but not by the European Medicines Agency (EMA). Approval was based on a phase III study showing that edaravone slowed the loss of physical function by 33% at 24 weeks compared with placebo on the ALSFRS-R scale [3]. However, the efficacy of the treatment remained controversial due to the short duration of the study and the strict inclusion criteria, which were limited to patients with an early stage of the disease. Only the significant improvement in the symptomatic treatment of ALS had a significant impact on patient survival and quality of life [4]. In particular, the development of non-invasive ventilators and the improvement of bronchial suction techniques have placed the management of ALS in the context of multidisciplinary care [5].

In this opinion view paper, we will discuss how to address some challenges for drug development in ALS, whether they are at the conceptual, technological, methodological, economic, or ethical levels. Finally, we will take a more optimistic view with the recent approval of two new drugs in some countries and the development of gene-targeted therapies that have raised hope in the ALS community and among patients.

## 2. Some Aspects of ALS Disease Mechanisms Acted upon by Therapeutic Strategies

**Figure 1:**
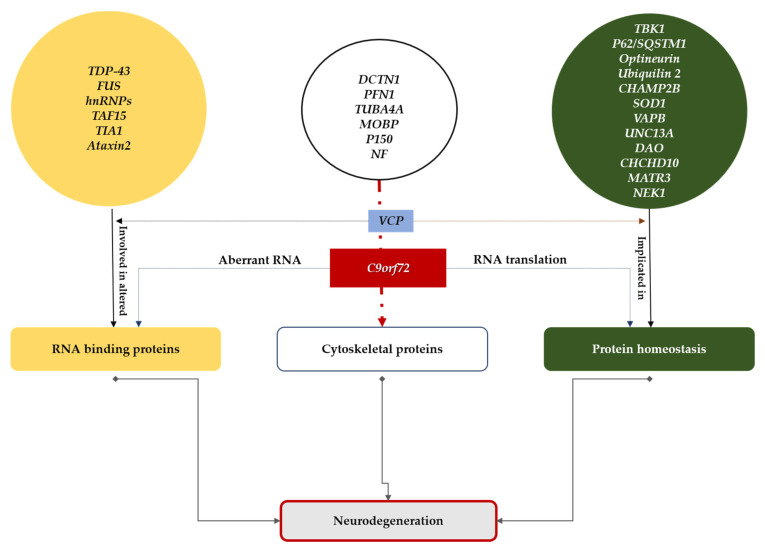
ALS mutations are involved in RNA metabolism, protein degradation, autophagy, or axonal transport.

This question arises to address the complexity of ALS resulting from the biological, genetic, and phenotypic heterogeneity of the disease [6,7,8]. Various strategies are currently being developed. A non-exhaustive list is given in Table 1. Since the identification of the first ALS -related gene in 1993, *SOD1* [9], more than 40 genes have been associated with ALS pathological mechanisms. These mutations are responsible for about 65% of familial ALS and 10% of sporadic ALS. Four major genes (*C9ORF72, FUS, TDP-43, SOD1*) are found in about 80% of familial ALS cases. The most common gene is a non-coding G4C2 hexanucleotide repeat expansion in the *C9ORF72* gene, which is responsible for ALS, frontotemporal Dementia (FTD), or ALS /FTD, and accounts for about 30% of familial ALS and 5% of sporadic cases [10,11,12]. In addition, the identification of rare genetic mutations in ALS has been crucial in determining the critical biological pathways underlying the degenerative cascade [13]. Several ALS mutations are present in genes whose products are involved in RNA metabolism, protein degradation [14], autophagy [15], or axonal transport [16]; some of these represent potential targets for future treatment strategies (Figure 1).

The discovery in 2006 that inclusions in motor neurons consist of aggregated TDP 43 protein that is mislocalized and phosphorylated in the cytoplasm of motor neurons was a breakthrough [37]. Several molecules under development modulate protein quality control mechanisms, for example, by stimulating the production rate of chaperone proteins to limit the conformational changes of mutant proteins (unfolding or misfolding) that are responsible for their tendency to aggregate [38]. Another strategy is to promote protein degradation through autophagy and proteasome activators [39]. Recent work has highlighted the central role of defects in nucleocytoplasmic transport, which may explain the cytoplasmic mislocalization of TDP-43 [40,41]. New approaches are therefore aimed at restoring molecular transport between the nucleus and the cytoplasm.

Several studies showed that motor neuron degeneration is a non-cell-autonomous process and have demonstrated that non-neuronal cells such as astrocytes, microglia, and oligodendrocytes directly contribute to motor neuron damage [42,43,44,45]. Dysfunction of the astrocyte-expressed EAAT2 transporter, which uptakes the neurotransmitter glutamate from the synaptic cleft, is reduced in the cortex and spinal cord of ALS patients, which may be related to motor neuron excitotoxicity [44]. The role of microglial activation, which occurs in ALS as in many other neurodegenerative diseases, has received considerable attention in animal models, but its effects in ALS patients are poorly documented [42,46,47,48,49]. Whether the activation of microglia is advantageous or disadvantageous for the motor neurons, however, remains an unresolved question [50,51]. In the context of therapeutic development, it is imperative to distinguish harmful from neuroprotective mechanisms to define a therapeutic window for treatment initiation.

Beyond the cells in the nervous system, other cells involved in the non-cell autonomous degeneration of the CNS are also of great interest. The role of peripheral inflammation has been outlined with the role of T lymphocytes and monocytes/macrophages [52,53,54,55]. Several studies have reported activation of monocytes in the peripheral blood of ALS patients [56] and increased invasion of peripheral monocytes into the spinal cord of ALS patients and mice [57,58], contributing to motoneuron loss. Studies in ALS patients have shown that there is an association between reduced numbers of Tregs and increased disease severity, progression, and survival [59,60]. Finally, alterations in the muscle and neuromuscular junction may also play a role in retrograde degeneration of the motoneuron [61,62], with changes in gene expression in the muscle being associated with disease progression [63]. Several mechanisms have been proposed, such as increased expression of proteins such as Nog-A that inhibit neuromuscular stabilization [64], satellite cell abnormalities [65], a reduction in trophic factors secreted by the muscle [66], or, more recently, the secretion of toxic exosomes [67].

## 3. Therapeutic Approaches

### 3.1. Pharmacologic Approaches

Table 1 provides a non-exhaustive list of ongoing clinical trials to illustrate the variety of different disease mechanisms being investigated in this complex and multifactorial disease. Recently, two drugs have been approved in some countries. The IV formulation of the drug (Radicava^TM^) had previously been approved by the FDA in 2017. The approval was based on a phase III study that showed edaravone slowed the loss of physical function by 33% after 24 weeks compared to placebo on the ALSFRS-R scale [3]. However, the effectiveness of the treatment remained controversial, and it was not approved by EMA. One reason for this was the short duration of the trial and the strict inclusion criteria, which were limited to patients in the early stage of the disease. The high level of care required with repeated daily IV infusions was a limitation for prescribing the drug. This led to the development of an oral form of edaravone (Radicava ORS^TM^) by Mitsubishi Tanabe Pharma, which was recently approved by the FDA in May 2022, and a phase III trial is currently underway to assess the long-term safety and tolerability of oral edaravone over 96 weeks (NCT04577404). The second drug is from Amylyx Pharmaceuticals, an oral, fixed-dose co-formulation of sodium phenylbutyrate and ursodoxicoltaurine (Albrioza^TM^) to address both mitochondrial dysfunction and endoplasmic reticulum stress. In June 2022, the co-formulation received its first conditional approval in Canada for the treatment of (ALS) in adults. The approval was based on the results of the multicentre phase II CENTAUR trial (NCT03127514), in which the slowing of progression of ALS with the treatment compared with placebo [20].

Another therapeutic approach that has recently gained renewed interest is the targeting of muscle abnormalities in ALS [68]. The first rationale is neuroprotective, as changes in the muscle and neuromuscular junction may play a role in retrograde degeneration, as suggested by our recent work on the role of secretion of toxic exosomes by muscle in motor neuron degeneration [67]. A second approach is symptomatic by increasing muscle contractility, with two troponin activators in development, tirasemtiv [24,25] and reldesemtiv (NCT04944784), or improving muscle mass and strength [69].

### 3.2. Gene and Cell Therapy Approaches

Recently, experimental strategies targeting genes have come to the forefront of clinical research, offering the promising therapeutic potential for ALS and hope for patients with ALS. Several technologies are being tested in preclinical or clinical phases. These include antisense oligonucleotides (ASO), interfering RNAs, viral vectors, or gene editing with CRISPR/Cas9 [70]. Successful treatment with an ASO (Nusinersen^TM^) and then with a viral vector (Zolgensma^TM^) for another motor neuron disease, spinal muscular atrophy (SMA), has raised hopes that these approaches will lead to approved drugs for ALS in the short term. It should be emphasized, however, that in autosomal dominant forms of ALS, it is no longer a question of compensating for the loss of function of a deleterious gene, as is the case with SMA, but on the contrary of decreasing the expression of a mutation that leads to a toxic gain of function. Such approaches are currently being tested in clinical trials for patients with mutations in *SOD1*, *C9ORF72*, and *FUS* genes.

The most advanced ASO-based treatment is Tofersen^TM^, developed by Biogen, which is designed to reduce the synthesis of the *SOD1* protein [35,71]. The VALOR study enrolled 108 ALS patients with an *SOD1* mutation who were treated for 28 weeks (NCT02623699). The main results were disappointing, as the main objective of slowing functional deterioration as measured by the ALSFRS-R was not achieved [71]. However, Tofersen^TM^ will apply for approval under the accelerated approval process based on data showing a marked decrease in neurofilament light (NfL) levels, a biomarker of neuronal degeneration, and a reduction in *SOD1* protein associated with a trend towards less disease progression [71]. Based on the reasonable assumption that treatment is more effective at an early stage of degeneration, a clinical trial has recently been started in subjects who are carriers of the *SOD1* mutation and do not yet have clinical manifestations of the disease (ATLAS study, NCT04856982). The study is investigating whether Tofersen^TM^ can delay the onset of the disease [72,73]. Subjects are eligible for intervention in this study if the follow-up of the subjects reveals an increase in NfL levels in the blood above a certain threshold that has been shown to predict the onset of symptoms within one to two years [73,74].

A study has been conducted with another ASO-based treatment, Tadnersen^TM^ (BIIB078), which selectively inhibits the mutant *C9ORF72* transcripts [75,76]. Although the therapy was generally safe and well tolerated in people with *C9ORF72*-associated ALS, it did not result in significant clinical benefit compared with placebo. The extension study was stopped, and clinical development was discontinued. Wave Life Sciences is taking a similar approach with its investigational drug WVE-004, a stereopure ASO, targeting variants containing G4C2, a hexanucleotide repeat expansion associated with the *C9ORF72* gene. This study, the phase Ib/IIa FOCUS -C9 trial (NCT04931862), was initiated in August 2021 and is evaluating WVE-004 in *C9ORF72*-associated ALS and frontotemporal dementia. A phase III trial of Jacifusen^TM^ (NCT04768972), an ASO designed to reduce *FUS* protein synthesis from *FUS* mRNA, is ongoing for patients with *FUS* gene mutations associated with aggressive juvenile forms of ALS [77,78]. In contrast to ASOs targeting inherited forms of ALS, other strategies are currently being developed that are applicable to sporadic cases and aim to modulate the expression of disease-modifying genes. A phase I trial of BIIB105, an ASO targeting the ataxin-2 gene, is currently underway in sporadic patients with ALS (NCT04494256) [79]. The first rationale is that polyglutamine expansions in ataxin-2 increase the risk of ALS in people who carry them. Secondly, work in yeast and fly models has shown that ataxin-2 promotes aggregation and toxicity of the TDP-43 protein [80].

However, recent evidence has raised awareness that while these strategies will certainly diversify, the challenge of effectiveness and safety remains significant. These risks should be considered and are clearly underscored by the failure of a trial of the ASO Tominersen^TM^ in Huntington’s disease, where the trial was stopped prematurely because the participants’ symptoms worsened [81]. A major concern with ASO treatment is that the treatment that aims to decrease the levels of the abnormal protein also affects its normal counterpart and, therefore, its physiological function. Among ALS causal mutation, this concern is important with *C9ORF72* mutations; whether the mechanism is loss and/or gain of function remain controversial [82]. Strategies based on genome editing, in particular CRISPR/Cas9 technology, could specifically target the genetic mutations, such as removing the intronic position of the *C9ORF72* repeat expansion by a ‘cutting-deletion-fusion’ method [11,83,84]. A second risk is the potential immunogenicity of ASO and the risk of meningitis when administered intrathecally. Serious neurological events were reported in 4.8% of ALS patients receiving Tofersen^TM^, including two cases of myelitis (2.0%).

The therapeutic approach using stem cells has recently been promoted as a potential neuroprotective therapeutic strategy for ALS. In particular, mesenchymal stem cells (MSCs) have multiple effects, such as stimulation of intrinsic neurogenesis, the release of various neurotrophic factors, and modulation of immune-inflammatory processes, transforming the patient’s environment from a pro-inflammatory toxic state to an anti-inflammatory and neuroprotective state [85]. Several studies investigating the effect of therapeutic approaches using MSCs in mouse disease models have shown that motor neuron loss was slower in the group treated with MSCs [86,87,88,89,90,91]. Subsequently, several clinical trials were conducted to investigate the therapeutic effect of MSCs in ALS patients using intrathecal or intraspinal administration of bone marrow-derived mesenchymal or mononuclear cells or fetal neural stem cells [92,93,94,95]. However, a recent phase III trial of intrathecal administration of MSCs in ALS patients did not meet its primary endpoint of a change in ALS decline, although participants with less severe disease retained more function compared with the placebo group (NCT03280056) [96]. It shows that there is still much to be done in terms of the source of stem cells, the mode of administration, the selection of potentially better-responding patients, clinical endpoints, and safety [97,98,99,100,101,102].

## 4. Preclinical and Clinical Development

### 4.1. Improve Preclinical Models

Some of the disease models used in recent years have been questioned. This is mainly because none of the studies conducted were able to translate the results from the animal model well to ALS patients. Furthermore, the only approved neuroprotective treatment, riluzole, which showed a positive effect in ALS patients, showed no effect in a mouse model [103]. One of the most important animal models for diseases in ALS is the SOD1 mice model, transgenic mice expressing a mutation in the SOD1 gene are undoubtedly important for understanding the biological mechanisms of ALS [49]. However, this model has an inherent limitation in that it represents only 10% of familial ALS, which in turn represents 10% of all ALS cases, and it does not represent an important pathological feature of the disease, TDP-43 accumulation [103,104]. Moreover, the failure was at least partly due to methodological errors. Therefore, there are increasing recommendations to improve preclinical design, validate animal models of disease and encourage the publication of negative results [105,106,107,108,109,110,111,112,113,114,115,116]. The lack of a validated model for the sporadic form of the disease remains a real obstacle. Therefore, considerable efforts have been made to develop cellular disease models from human-induced pluripotent stem cells (hiPCS) [117]. These in vitro models allow for easy modeling of the disease and screening of drug candidates, including already approved drugs, which allow the repositioning of drugs in ALS [118]. Interestingly, hiPCS-based techniques enabled the identification of several therapeutic drug candidates in ALS, such as ropinirole (a dopaminergic agonist used in Parkinson’s disease), retigabine (activator of voltage-gated potassium channels used as an anticonvulsant) and bosutinib (src tyrosine kinase inhibitor used to treat chronic myeloid leukemia), and some of these are currently being investigated in clinical trials [119]. Furthermore, from a precision medicine perspective, it may well be possible to select the most appropriate treatment for a patient based on its effect on his or her own iPCS [120].

### 4.2. Design of Clinical Trial in ALS

**Figure 2:**
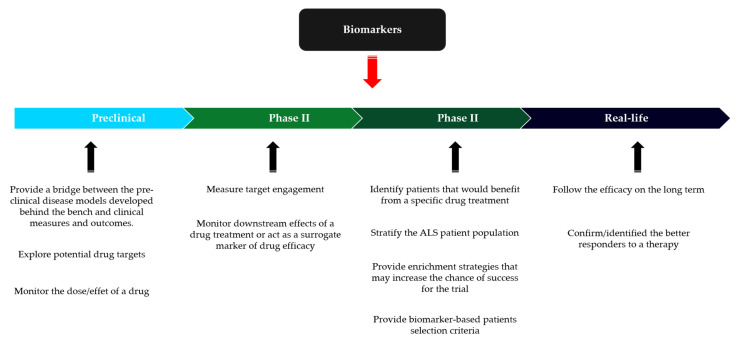
Summaries the utility of biomarkers in drug development in ALS spans from preclinical models, with translational biomarkers, to real-life studies.

Consideration of the heterogeneity of ALS has led to a reassessment of the design of therapeutic trials. There have been notable developments in enrichment strategies based on the selection and stratification of patients who are more likely to show a better response to treatment in trials. They are based on prognostic clinical variables such as the rate of disease progression, diagnostic delay, or biological characteristics such as a causative genetic mutation or a predictive biomarker profile [121]. These approaches are now more widely accepted in FDA guidelines [122], but they inevitably limit extrapolation of their efficacy to heterogeneous populations found in real life. An example is edaravone, which was approved by the FDA, although modest efficacy was found only in certain forms diagnosed at an early stage and not very severely impaired [3]. The development of a validated biomarker is currently one of the most active research areas on ALS. Several studies focus on the development of diagnostic, prognostic, or predictive biomarkers that could partially explain some of the reasons for heterogeneity, define different ALS biotypes, help stratify patients and facilitate the prediction of subgroups of patients who respond to treatment [123,124,125]. The utility of biomarkers in drug development in ALS spans from preclinical models, with translational biomarkers, to real-life studies (Figure 2). Several candidate biomarkers have been explored, such as biological biomarkers (neurofilaments in cerebrospinal fluid (CSF) and plasma [126], miRNAs), neurophysiological (Motor Unit Number Estimation (MUNE), Motor Unit Number Index (MUNIX) [127], electrical impedance myography (EIM), transcranial magnetic stimulation (TMS) [128]), neuroradiological (diffusion tensor imaging, functional MRI, iron-sensitive sequences, voxel-based morphometry [129]), and digital (accelerometry, quantified acoustic analysis of dysarthria [130]). It is hoped that these biomarkers will replicate the effective development process achieved in multiple sclerosis thanks to MRI, which has allowed sufficiently promising therapeutic molecules to be selected in short phase II trials [131,132,133]. However, it is necessary to draw a lesson from therapeutic developments in Alzheimer’s disease. While anti-amyloid antibodies showed impressive efficacy on amyloid plaque burden in the PET scan, they showed no clinically significant or at best marginal effect in the phase III clinical trial [134].

### 4.3. Economic and Ethical Realities

The importance of having validated evidence through phase III trials is not easily reconciled with economic realities, as the costs of this type of study limit the number of promising treatments that will reach this phase of development, and ethical realities, due to the legitimate demand of patients for rapid access to new treatments, and with the ethical aspects due to the legitimate demand of patients to have new treatments quickly. One of the answers is to develop platforms for therapeutic trials, such as the Healey platform coordinated by Massachusetts General Hospital [135]. The concept is to test multiple molecules in parallel and adaptively by pooling groups treated with a placebo. According to the developers, this FDA-supported platform could cut the time to market approval of treatment by half and the cost by at least one-third. The issue of timely access to treatments for rare diseases and serious prognoses also arises in a societal context, such as right-to-try, which is promoted by certain patient advocacy groups. It prompts drug authorities to think about early access and conditional approval programs that are subsequently validated in phase III or real-world settings.

## 5. Conclusions

There are positive signs of new treatments for ALS, especially the hope raised by the advent of targeted gene therapies and the recent approval of two new drugs in some countries. Full stabilization of the disease and especially regenerative therapies are still part of a longer-term perspective. Treatments that are not conventional approaches to neuroprotection, such as those that act on the microbiota, or nutritional interventions that target the metabolic disturbances observed in the disease, also need to be considered. This multifactorial character at the biological level brings us back to an old view in the field of neurodegeneration, which assumes that only the combination of several treatments can achieve a real clinical effect.

## Figures and Tables

**Table 1:** A non-exhaustive list of therapeutic agents in development for ALS.

Agent	Targeted Mechanism	Mechanism	Results	Phase	Ref.
Sodium Phenylbutyrate-Taurursodiol	endoplasmic reticulum stress, and mitochondrial dysfunction	Sodium phenylbutyrate is a histone deacetylase inhibitor that has been shown to upregulate heat shock proteins and act as a small molecule chaperone, alleviating endoplasmic reticulum stress toxicity [17,18]. Taurursodiol recovers mitochondrial bioenergetic deficits through multiple mechanisms, including preventing the translocation of Bax protein into the mitochondrial membrane, thereby decreasing mitochondrial permeability and increasing the cell’s apoptotic threshold [19]	Less functional deterioration measured by the ALSFRS-R score over a 24-week period. Secondary outcomes, including decreases in isometric muscle strength and vital capacity, did not differ significantly between groups	II	[20]
Colchicine	Protein aggregates, autophagy, and neuroinflammation	Colchicine could upregulate proteins involved in autophagy, including the TFEB, the TFEB-regulated adaptor protein SQSTM1/p62 and the autophagy player microtubule-associated protein 1A/1B-light chain 3 (LC3).	Ongoing	II	[21]
Rapamycin	Autophagy and neuroinflammation	Rapamycin is based on the inhibition of mTORC1. mTORC1 targets regulatory proteins in cell signalling and regulates autophagy by inhibiting the unc-51-like kinase 1 complex.	Ongoing	II	[22]
BIIB100 (KPT-350)	Nucleocytoplasmic transport dysfunction	Selective inhibitor of nuclear export that inhibits exportin 1 (XPO1; CRM1).	Ongoing	I	
Deferiprone	Iron accumulation	Iron Chelation	Ongoing	II	[23]
TIRASEMTIV	Muscle contractility	A FSTA that selectively activates the fast skeletal muscle troponin complex by increasing its sensitivity to calcium	In a phase IIb clinical trial, SVC and muscle strength were found to decline significantly more slowly in tirasemtiv-treated participants. But no significant difference was found in the decline in functional disability as measured by the ALSFRS-R. However, no significant difference in disease progression was demonstrated in the phase III clinical trial.	II/III	[24,25]
Interleukine 2	Neuroinflammation	Immunomodulatory strategy by promoting Treg expansion, which attenuates neuroinflammation.	A phase IIa study showed that low dose IL-2 is well tolerated and immunologically effective in subjects with ALS [26]	III	[26]
Masitinib	Neuroinflammation	Tyrosine kinase inhibitor targets microglia and mast cells through inhibiting a limited number of kinases. Masitinib blocks microglia proliferation and activation, and mast cell-mediated degranulation, the release of cytotoxic substances that might further damage the motor nerves.	A randomised, placebo-controlled phase III trial has previously shown that oral masitinib (4.5 mg/kg/day) slows the rate of functional decline with acceptable safety in ALS patients with an ALSFRS-R progression rate of <1.1 points/month	III	[27]
Ibudilast (MN-166)	Neuroinflammation	Inhibitor of macrophage migration inhibitory factor and phosphodiesterases 3,4,10 and 11 [28,29]. Ibudilast attenuates CNS microglial activation and secretion of pro-inflammatory cytokines.	Ongoing	II/III	[29,30]
Fasudil	Neuroinflammation	Rho kinase inhibitor	Ongoing	II	[31]
Ravulizumab	Neuroinflammation	Humanized monoclonal antibody to complement factor 5 which acts to block complement activation	The independent Data and Safety Monitoring Board monitoring committee recommended that the study be discontinued due to lack of efficacy. No new safety findings were observed.	III	[32]
Zilucoplan	Neuroinflammation	A small molecule that works aa s C5 complement inhibitor	The The independent Data and Safety Monitoring Board recommended stopping the zilucoplan regimen because the likelihood of meaningfully slowing disease progression was considered low.	III	[33]
Anakinra	Neuroinflammation	The monoclonal antibody that works as a IL–1 receptor antagonist	Ongoing	II	
Tocilizumab	Neuroinflammation	The monoclonal antibody that works as a IL–1 receptor antagonist	Tocilizumab is safe and tolerable and reduces C-reactive protein concentrations in the plasma and cerebrospinal fluid of ALS patients	II	[34]
Tofersen (BIIB067)	Gain of function *SOD1*	It is an antisense oligonucleotide (ASO) targeting *SOD1*	In the Phase III VALOR study, the primary endpoint as measured by the ALSFRS-R did not reach statistical significance; however, signs of reduced disease progression across multiple secondary and exploratory endpoints were observed	III	[35]
BIIB078	Gain of function *C9ORF72*	It is an antisense oligonucleotide (ASO) for *C9ORF72*-associated ALS	In a Phase I study, BIIB078 was generally well-tolerated. The adverse events were mostly mild to moderate in severity and occurred at a similar rate across BIIB078 and placebo groups. BIIB078 did not meet any secondary efficacy endpoints and it did not demonstrate clinical benefit. Therefore, the clinical program will be discontinued	I	[36]

Abbreviations: ALSFRS-R, ALS Functional Rating Scale-revised; FSTA, fast skeletal muscle troponin activator; SVC, slow vital capacity; TFEB, master regulator transcription factor EB.
